# Engineering immunity with CAR-NK cells: advancing the frontiers of cancer immunotherapy

**DOI:** 10.3389/fphar.2025.1738558

**Published:** 2025-12-19

**Authors:** Vlad Andrei Cianga, Ion Antohe, Cosmin Minciună, Angela Dăscălescu

**Affiliations:** 1 Hematology Department, Regional Oncology Institute, Iasi, Romania; 2 Grigore T Popa University of Medicine and Pharmacy Iasi, Iasi, Romania; 3 Bone Marrow Transplant Department, Regional Oncology Institute, Iasi, Romania

**Keywords:** CAR-NK cell therapy, immunotherapy, adoptive cell immunotherapy (APC), CAR engineering, ICANS, immune effector cell-associated neurotoxicity syndrome, CRS, cytokine release syndrome

## Abstract

Chimeric antigen receptor–modified natural killer (CAR-NK) cells are emerging as a promising alternative to CAR-T therapies, offering advantages such as reduced toxicity, allogeneic feasibility, and flexible manufacturing. Current reviews cover NK biology and CAR engineering progress, yet lack a unified perspective that connects these advances. This review provides a novel synthesis by mapping specific tumor immune evasion mechanisms, including antigen loss, lineage plasticity, impaired antigen processing, epitope masking, and trogocytosis to corresponding next-generation CAR-NK engineering solutions. This “evasion-to-solution” framework highlights how innovations such as dual-antigen CARs, low-affinity designs, NK-specific signaling, iPSC-derived NK platforms, and multiplex gene editing directly mitigate known mechanisms that lead to therapeutic failure. By linking tumor biology to engineering strategy, this review offers a translational roadmap for the rational design of more adaptable and resilient CAR-NK therapies.

## 1 Introduction

Cancer immunotherapy is rapidly advancing, with new approaches leveraging the immune system to target tumors. Gene manipulation has led to adoptive cell therapies (ACT), providing promising treatments for blood and solid cancers when other options fail. CAR-T and CAR-NK cells are genetically engineered to boost anti-tumor responses (Neelapu et al., 2017; Maude et al., 2018; Park et al., 2018; June et al., 2018; Schuster et al., 2019; Wang et al., 2020; Melenhorst et al., 2022). CAR T-cell therapies have revolutionized the treatment of hematological malignancies (Mitra et al., 2023). CD19-targeted CAR-T cells are now standard for B-ALL and lymphomas, and BCMA-targeted CARs show promise in multiple myeloma. Despite their benefits, CAR-T therapies are hindered by high costs, complex manufacturing, and limited access due to their autologous design (June et al., 2018; Roex et al., 2020; Borgert, 2021; Cutmore and Marshall, 2021). Additionally, toxicities like cytokine release syndrome (CRS) and immune cell neurotoxicity (ICANS) can cause significant health risks (Hunter and Jacobson, 2019). These limitations have prompted research into safer, more accessible options. Natural killer (NK) cells, with their tumor-targeting abilities and lower risk of secondary toxicities, are emerging as promising candidates for next-generation CAR-based therapies (Romee et al., 2016; Rezvani et al., 2017; Liu et al., 2020; Daher and Rezvani, 2021). Most reviews on CAR-NK cell therapy cover clinical trial updates, production methods, and immunological advantages over CAR-T therapies (Smith et al., 2020). However, they typically treat tumor biology and CAR-NK engineering separately, without integrating tumor immune evasion with CAR design improvements. This review introduces a conceptual framework linking tumor escape mechanisms to specific CAR-NK engineering strategies. Tumors evade immune detection through antigen loss, MHC class I downregulation, trogocytosis, and immunosuppressive signals, each presenting unique design considerations. For instance, dual-target CARs address antigen escape, affinity tuning helps manage trogocytosis, NK therapies leverage HLA downregulation, and armored CARs counteract suppressive tumor environments (Zhong and Liu, 2024). This review connects tumor evasion mechanisms to new CAR-NK strategies, such as personalized co-stimulatory domains, gene editing, and iPSC-derived platforms, creating a clear roadmap for therapy development. The proposed framework guides the design of CAR-NK therapies for both blood and solid cancers, aiming to support the creation of robust, flexible, and safe allogeneic treatments.

## 2 The immune biology of NK cells

### 2.1 Ontogeny

Natural killer (NK) cells, a type of innate lymphoid cell (ILC), are crucial for host defense by providing rapid cytotoxic responses through germline-encoded receptors rather than peptide–MHC recognition like CD8^+^ T cells (Chiossone et al., 2018). Originating from CD34^+^ hematopoietic stem cells in the bone marrow, NK cells mature in secondary lymphoid tissues and undergo a licensing process in the thymus (Freud and Caligiuri, 2006; Stokic-Trtica et al., 2020). Their migration and tissue distribution depend on chemokines, integrins, and selectins: CX3CR1 and CXCR4 regulate bone marrow exit, while CCR7-CCL19 directs organ-specific homing (Pesce et al., 2016; Ran et al., 2022). This enables NK cells to monitor multiple tissues, including the liver, lungs, intestine, uterus, and CNS (Moroso et al., 2011; Vacca et al., 2013).

### 2.2 Maturation and differentiation

NK cell maturation is marked by morphological and phenotypic changes regulated chiefly by transcription factors EOMES and T-bet, influencing cytotoxicity and tissue residency (Abel et al., 2018; Kiekens et al., 2021). Cytokines like IL-12, IL-15, and IL-18 promote maturation and memory-like traits with stronger responses upon restimulation (Romee et al., 2012). Single-cell transcriptomics show diverse NK phenotypes, including terminally differentiated, memory-associated subsets (Smith et al., 2020). NK cells are identified by CD56 expression. Functional subsets are classified mainly by CD56 and CD16 surface density: CD56^bright^CD16^dim^/^−^ cells secrete cytokines and reside in tissues, while mature, lytic CD56^dim^CD16^+^ cells are predominant in blood (Hu et al., 2019; McErlean and McCarthy, 2024). Though considered less mature, CD56^bright^ NKs express NKG2 receptors and proliferate in response to IL-2 and IL-15. CD56^dim^ NKs that express KIRs (killer immunoglobulin-like receptors) are highly cytolytic. CD57 expression marks terminal differentiation and immunologic memory capability (Stabile et al., 2017; Liu et al., 2021).

### 2.3 Functionality

NK cells, unlike classic lymphocytes, use somatically encoded receptors for activation and cytotoxicity rather than receptor recombination (Marin et al., 2024). They detect tumor cells with reduced MHC expression and act through cytokine secretion and direct killing mechanisms like ADCC (antibody dependent cell cytotoxicity), Fas-mediated lysis, and TRAIL-mediated apoptosis (Smyth et al., 2001). NK cells also produce cytokines such as IFN-γ (interferon-gamma) and TNF-α (tumor necrosis factor alpha), promoting inflammation and activating other immune cells (Vivier et al., 2011; Fauriat et al., 2010; Wang et al., 2012). CD16 enables recognition of IgG-coated targets, triggering ITAMs and intracellular signaling for ADCC (Biassoni and Malnati, 2018). Signaling leads to degranulation and apoptosis in target cells via perforin, granulysin, and granzymes (Prager and Watzl, 2019). The diversity of NK cell functions is linked to their maturation, phenotype, and location, offering insights into overcoming tumor immune evasion (Capuano et al., 2021).

NK-cell research has shifted from basic immunobiology to translational engineering, focusing on their pharmacological profile as living drugs. Understanding NK cell maturation, activation, and cytokine needs is crucial for optimizing design and manufacturing. This review links biological mechanisms to next-generation engineering decisions that drive CAR-NK clinical outcomes.

## 3 CAR-T limitations due to tumor immune evasion strategies

Cancer cells employ various tumor escape mechanisms (TEMs) to avoid immune recognition. For example, many malignancies downregulate MHC class I molecules to escape T cell detection. NK cells, by contrast, are less susceptible to such mechanisms and can attack cells with low MHC-I by sensing stress-induced ligands. Key activating receptors on NK cells include the natural cytotoxicity receptors (NCRs) and NKG2D, whereas inhibitory receptors (KIRs and CD94/NKG2A) counterbalance activation and enforce self-tolerance. The interplay of these signals ultimately determines NK cell activity (Lanier, 2015; Sivori et al., 2019; Yi et al., 2023). Importantly, fundamental differences in target recognition between T cells and NK cells mean that the tumor microenvironment drives diverse relapse mechanisms in CAR-T therapy. Even though the innate properties of NK cells provide some intrinsic resilience, novel specific engineering solutions are needed to efficiently overcome tumor escape and improve patient outcomes (Gu et al., 2022; Jørgensen et al., 2025). In the following subsections, we address key intrinsic tumor immune evasion strategies and extrinsic microenvironmental barriers and, also, discuss how CAR-NK cell design is currently optimized to counter each challenge.

### 3.1 Antigen-negative tumor clones

Pre-existing antigen-negative tumor subclones (e.g., CD19^−^ or CD22^−^ malignant B cells), or the loss of a target antigen under CAR-T selection pressure, can lead to therapeutic resistance. A retrospective analysis of 628 B-ALL patients found that approximately 17% harbored CD19-negative and 22% had CD22-negative leukemic clones prior to CAR-T therapy (Rosenthal et al., 2018; Lin et al., 2024). This indicates that antigen-loss variants can exist at baseline and, upon CAR-T treatment, these antigen-negative cells can outgrow the targeted tumor population, causing relapse via immune escape. CAR-NK cells, while also susceptible to this escape, however, retain additional innate cytotoxicity pathways that can kill tumor cells beyond CAR recognition. This means CAR-NK cells are capable of partially eliminating antigen-negative tumor cells via natural NK receptors, an advantage over conventional, antigen-restricted, CAR-T cells (Jørgensen et al., 2025). Nevertheless, complete loss of the target antigen poses a major challenge, and preventing the escape of antigen-negative clones remains a priority in CAR-NK engineering.

### 3.2 Antigen mutations and alternative splicing

Another cause of CAR-T ineffectiveness is genetic alteration of the target antigen itself. Mutations or alternative mRNA splicing in the antigen’s gene can downregulate its expression or generate variant antigens, effectively “shielding” the tumor from CAR binding (Lin et al., 2024). In one study of 12 B-ALL patients who relapsed after anti-CD19 CAR-T therapy, each patient had a unique insertion or deletion in the CD19 gene (most frequently affecting exons 2–5). These mutations led to truncated CD19 proteins missing the CAR recognition epitope, thereby enabling immune evasion. Similarly, alternatively spliced CD19 transcripts that skip exons (for instance, loss of exon 2) result in a truncated protein or an isoform lacking the CAR-binding domain, rendering the CAR-T cells ineffective (Fischer et al., 2017; Orlando et al., 2018; Bagashev et al., 2018).

### 3.3 Antigen processing and presentation defects

Defects in the intracellular processing or presentation of the target antigen can result in insufficient surface expression for effective CAR-T cells detection. For example, loss of critical chaperone proteins or disruptions in antigen trafficking may prevent the antigen from reaching the cell surface. Tumor cells may also evade CAR-T cells by internalizing the target receptor or sequestering it in subcellular compartments, effectively “hiding” the antigen from immune surveillance (Lin et al., 2024).

### 3.4 Lineage switch

Lineage switching is a rare but striking mechanism of immune escape wherein the tumor changes its cellular lineage identity. This phenomenon, observed mostly in B-ALL treated with CD19-targeted CAR-T cells or monoclonal antibodies, involves leukemic cells shifting from a lymphoid lineage to myeloid lineage. Such a transition can occur via transcriptional reprogramming or the emergence of a distinct leukemic subclone (Kurzer and Weinberg, 2022; Coorens et al., 2023; Lin et al., 2024). As a result, CD19^+^ B-ALL can relapse as CD19^−^ acute myeloid leukemia (AML), escaping CAR-T recognition. In fact, several cases of lineage switch have been reported post-CAR therapy: chronic lymphocytic leukemia transforming into plasmablastic lymphoma (Evans et al., 2015), mantle cell lymphoma transforming into histiocytic sarcoma (Zhang et al., 2020), and T-ALL transforming into AML after CD7 CAR-T treatment (Aldoss et al., 2023). This extreme plasticity allows tumors to evade lineage-specific therapies (like anti-CD19 CAR-T) by evolving into different tumors.

### 3.5 Epitope masking

Although uncommon, epitope masking is a particularly insidious mechanism of CAR-T failure. In a case reported by Ruella et al. (2018), an accidental transduction of a leukemic B cell with the CD19 CAR construct caused the tumor cell to express the CAR on its own surface. The CAR on the tumor bound to its own CD19 antigen, effectively concealing CD19 from therapeutic CAR-T cells. This self-masking of the target antigen rendered the CAR-T treatment ineffective. Consequently, strict quality control during CAR-T manufacturing is essential to prevent accidental tumor cell transduction. Moreover, the use of allogeneic “off-the-shelf” CAR-T or CAR-NK cell products (derived from healthy donors) could avert this complication entirely, since tumor cells would not be present in the engineered cell product (Ruella et al., 2018; Lin et al., 2024).

### 3.6 Antigen-mediated trogocytosis

Trogocytosis, a process of membrane exchange between cells, can also drive antigen escape. CAR-T cells can strip target antigen from tumor cells by trogocytosis, reducing antigen density on the tumor cells and thereby building resistance to further CAR-T engagement. This phenomenon contributes to CAR-T cell exhaustion and cellular fratricide, as they may present acquired tumor antigens on their own surface. In some situations, trogocytosis can also, paradoxically, enhance tumor cell survival and migration by transferring certain immune molecules to tumor cells, allowing them to acquire immune-like features and better adapt within the tumor microenvironment (Hamieh et al., 2019; Lin et al., 2024). An in-depth analysis of trogocytosis-related effects is explored in subsequent sections of this review.

While the above escape mechanisms have been well-characterized in the context of CAR-T therapy, similar challenges may arise, to a lesser extent, with other adoptive cell therapies. CAR-NK cells share some vulnerabilities with CAR-T cells, especially when tumors evade by modulating the target antigen. Tumor-intrinsic resistance, coupled with limitations of CAR designs, can significantly impede the efficacy of these therapies. For example, the high genomic instability of cancer cells means that antigen downregulation could diminish CAR-NK cell effectiveness (Zhong and Liu, 2024).

Collectively, these immune evasion mechanisms not only drive resistance to CAR-T therapy but also provide critical insights for next-generation CAR-NK development. NK cells can recognize targets independently of MHC class I, making them less vulnerable to the loss of MHC-I or certain antigen-downregulation strategies. However, even CAR-NKs could be hindered by trogocytosis-mediated antigen loss, which reduces target density on tumor cells (Abel et al., 2018). Therefore, emerging CAR-NK design optimizations are being geared toward overcoming these escape mechanisms, as discussed next.

### 3.7 Engineering strategies to counter antigen escape and broader immune evasion mechanisms

To address antigen loss and tumor escape, researchers are developing several CAR engineering strategies (many inspired by advancements in CAR-T cells). These strategies aim to prevent or circumvent tumor antigen escape and improve the durability of responses.

#### 3.7.1 Inhibitory CARs (iCARs)

One approach is to incorporate negative regulators into CAR designs. For example, chimeric receptors built with checkpoint domains (from PD-1 or CTLA-4) can act as “inhibitory CARs” that require dual signals for full activation. This allows T or NK cells to respond only when a tumor cell presents a combination of antigens, improving selectivity and reducing off-tumor toxicity (Fedorov et al., 2013). By integrating multiple inputs, iCAR-equipped cells can be programmed to spare healthy cells (that express tolerogenic antigens) while attacking malignant cells that express both antigens.

#### 3.7.2 Dual-target and multi-specific CARs

Simultaneously targeting more than one antigen can reduce the selective pressure that leads to tumor escape. Multi-specific CAR constructs have been designed to recognize two or more tumor antigens. In preclinical and early translational studies, dual-targeted CAR-NK cells have shown a lower incidence of escape variants compared to single-antigen CARs (Cichocki et al., 2022a; Cichocki et al., 2022b). The redundant targeting ensures that even if one antigen is lost or downregulated, the CAR-NK can still eliminate the tumor via the secondary antigen.

#### 3.7.3 Lower-affinity CAR constructs

Fine-tuning the binding affinity of the CAR can also mitigate antigen escape. Recent studies demonstrated that CAR T cells engineered with a lower-affinity scFv exhibit diminished trogocytosis of the cognate antigen (Olson et al., 2022). By not binding too tightly, these “low-affinity” CARs minimize the removal of antigen from tumor cells, thereby preserving target expression and avoiding premature antigen loss. This strategy can maintain sufficient antigen density for ongoing immune recognition, all while retaining potent cytotoxic function (Olson et al., 2022).

#### 3.7.4 Synthetic biology approaches - switchable/adaptable CAR systems

New CAR platforms decouple antigen recognition from T/NK cell activation using molecular adapters. For instance, leucine-zipper–based systems like SUPRA CARs (split, universal, programmable CARs) or ZipCARs use separate binding and signaling modules. Antibody-adapter CARs employ a soluble adapter that bridges the CAR to the tumor antigen. These switchable systems allow real-time control over targeting specificity, CAR cell activation level, and dosing by adding or removing adapters. These modular CAR designs allow for sequential or combined targeting of tumor antigens as their expression patterns change (Cho et al., 2018; McCue et al., 2022). This approach increases safety and adaptability, enabling the effector cell to be redirected toward another antigen or turned off temporarily when necessary.

#### 3.7.5 Logic-gated CAR circuits

Like synthetic biology processes, logic-gated CAR designs require Boolean conditions to trigger a full response. A prime example is the synNotch system, where recognition of an initial antigen activates the cell to express a second CAR against a different antigen (Roybal et al., 2016). In this two-step AND-gate, T cells, or potentially NK cells, can be engineered to only kill targets that sequentially display two antigens. More complex circuits have also been created. Linking multiple receptor signals by using various combinations of AND/OR/NOT gates, CARs were programmed to distinguish targets with a specific antigen profile. In one study, T cells equipped with a triple-input logic circuit could selectively eliminate cells expressing three target antigens while ignoring cells expressing only one or two of those markers (Williams et al., 2020). This high precision targeting significantly reduces off-target effects and damage to healthy tissues. Moreover, tumor escape becomes far more difficult, since malignant cells would have to lose multiple antigens simultaneously to evade detection.

Even though many of these innovative strategies were initially developed and tested in CAR-T systems, they provide a valuable blueprint for CAR-NK cell design. As the CAR-NK field continues to mature, engineering strategies directly address key vulnerabilities and integrate precision synthetic biology, multiplex gene editing, and universal iPSC-derived platforms. Incorporating such multi-faceted and adaptable approaches could improve efficacy and persistence, ultimately helping to overcome tumor resistance mechanisms that limit current therapies. In the following chapters, we describe how contemporary CAR-NK design strategies specifically target the evasion mechanisms identified here, forming a mechanistic “evasion-to-solution” continuum that underpins the translational rationale for emerging CAR-NK platforms.

## 4 Design and engineering of CAR-NK cells

The introduction of CAR-NK therapy signifies a potential “paradigm shift” in cancer immunotherapy, mitigating many of the side effects experienced with CAR-Ts (Xie et al., 2020; Raftery et al., 2023). In this context, “off-the-shelf” refers to allogeneic NK cell products that are manufactured in large batches from a renewable donor or iPSC source, cryopreserved, and stored for on-demand use, without requiring patient-specific cell collection or individualized production. This model enables rapid treatment availability and uniform product characteristics across patients (Heipertz et al., 2021). The novel designs and engineering advancements can potentially offer distinct benefits that address manufacturing costs and other limitations, as well as streamline the production process, with increased availability and patient access (Maalej et al., 2023). This is mainly due to the possibility of relying on allogenic NK sources. Unlike currently available commercial CAR-T products, NKs can be developed from a potentially unlimited number of donors without the risk of GVHD complications, which significantly improves the safety of the therapy (Zhang et al., 2022). There are multiple possibilities of harnessing NK cells for “off the shelf” use in CAR manufacturing, such as utilizing various NK cell lines or by apheresis of iPSC-NKs (Heipertz et al., 2021; Caruso et al., 2022). Swift availability could mean critical efficiency for patients with refractory and rapidly progressive disease. Moreover, CAR-NK cells have shown promising results in both solid and hematological neoplasms, which certifies that largely available NK sources could benefit a spectrum of pathologies.

Both CAR-T and CAR-NK models have, traditionally, used the same design, but, recently, more personalized constructs have emerged, which lead to generation of variable cytotoxic profiles and various cytokines (Kotanides et al., 2020). The characteristics of the aimed structure should ensure specificity for cancer cells or overexpressed tumor markers. Furthermore, the interaction should not involve MHC signaling (MacKay et al., 2020). Novel CAR design approaches are increasingly tailored to NK cell biology by including signal domains that align with specific NK cell activation pathways. While early CAR-NK constructs initially relied on domains such as 4-1BB/CD28 (intracellular signaling and cell activation), which are typically found in 2^nd^ and 3^rd^ generation CAR-T products, newer models integrate specific NK motifs for superior signaling, activation and cytotoxic functions (MacKay et al., 2020).

The molecular structure of CARs is comprised of three parts: the ectodomain, the transmembrane region and the endodomain.

### 4.1 Ectodomain

The ectodomain is made up of the single-chain fragment variant (scFv), which consists of a linker protein that unites a heavy and light chain and, also, of a hinge region, anchoring the ensemble to the transmembrane domain. This structure is designed specifically for cognate antigen recognition. Since different scFv are capable of binding different epitopes in the same protein, this domain can determine both the specificity and the function of the CAR-NK cell (Haso et al., 2013). Since the scFv is synthetic in nature, antigen specificity can be affected due to the changed connectivity of the VL (variable light chain) and VH (variable heavy chain) domains. Computational-assisted design of the scFv aids in configuring functional structures by assembling the amino acid sequence of the CDRs (complementary-determining regions) and making precision target engagement more effective (Thokala et al., 2016; Krokhotin et al., 2019).

### 4.2 Transmembrane region

The transmembrane (TM) region acts like an anchor for the CAR against the cell wall and further connects to the endodomain, the structure responsible for generating intracellular signaling (Gong et al., 2021). Therefore, the TM domain is a critical component due to its role in influencing the function and the activation potential of the CAR construct.

### 4.3 Endodomain

For the endodomain, recent CAR models utilize the CD3ζ chain signaling domain, which has 3 ITAMs (immunoreceptor tyrosine activation motifs) per CAR. These ITAMs, in turn, activate the Syk or ZAP70 tyrosine kinases and PI3-kinase signaling (Orr and Lanier, 2010). In other instances, signaling domains derived from NK-specific activating receptors (CD28, CD16, NKp44, NKp46, NKG2D, DNAM-1 and 2B4) have been used in NK-92 cell lines to enhance toxicity and optimize signaling pathways (Li et al., 2018). 2B4 is a co-stimulatory domain that is known for its role in NK cell anti-tumor effects through improvement of cytotoxic activity and cytokine release when compared to the typical 4-1BB counterpart (Xu et al., 2019). A signaling adaptor molecule, DAP12 (DNAX-activation protein 12), was also associated with greater anti-cancer roles compared to the more traditional CD3ζ (Imai et al., 2005). A study conducted by Ye Li et al. analyzed CAR constructs that aim to specifically enhance NK cell potency *in vivo* and *in vitro*. Their conclusions established that sCFv-NKG2D-2B4-CD3ζ (NK-CAR4) boasted superior cytotoxicity, expansion and persistence capabilities than their CAR-T homologue with the scFv-CD28-4-1BB-CD3ζ (T-CAR structure) (Li et al., 2018). Moreover, DAP10 and DAP12 molecules, signaling adaptor proteins involved in activation of Syk-vav1-Erk and NF-kB pathways, demonstrated superior roles in NKG2C and NKG2D receptor activation compared to CD3ζ (Topfer et al., 2015; Wang et al., 2020). New 3^rd^ and 4^th^ generation CAR-NK constructs aim to enhance the tumor penetration and function of these cells in the immunosuppressive microenvironment.

### 4.4 NK cell specific signaling strategies

More recent work has addressed NK specific signaling strategies. One approach adapts DNAM-1 (CD226)-based chimeric receptors, which can stabilize the immune synapse and enhance cytotoxic function when used as CAR signaling modules (Wu et al., 2015; Cifaldi et al., 2023; Focaccetti et al., 2022). Other approaches focus on unique activating receptors, such as NKp46 or NKG2D, and their appropriate adaptor pathways (DAP10/DAP12 or FcRγ), to better mirror native NK activation and limit tonic signaling (Wang et al., 2022; Zhang et al., 2024; Peng et al., 2024). Recent work has further advanced NK-specific CAR design through composite signaling domains that better recapitulate native NK activation circuitry. DNAM-1-CD3ζ chimeric receptors in human NK cells boost recognition and killing of PVR/Nectin-2^+^ solid tumors, an effect enhanced by Nutlin-3a–induced upregulation of DNAM-1 ligands (Focaccetti et al., 2022; Cifaldi et al., 2023; Cifaldi et al., 2024). In parallel, other CAR platforms are built to utilize NCR such as NKp30 and NKp46 fused to adaptor modules (CD3ζ, DAP10 or DAP12), which in preclinical models increase cytokine production and tumor lysis against ALL, ovarian carcinoma, osteosarcoma, prostate carcinoma and rhabdomyosarcoma and more closely mimic native NK signaling (Cifaldi et al., 2024; Hu et al., 2018; Yu et al., 2022; Wang et al., 2024). Together with newer multi-component endodomains that integrate NKG2D/DAP10, 2B4 and CD3ζ signaling to enhance activation (Hu et al., 2018; Yu et al., 2022; Yi et al., 2025), these architectures exemplify the rapid evolution of NK-tailored CAR backbones over the past few years.

**FIGURE 1:**
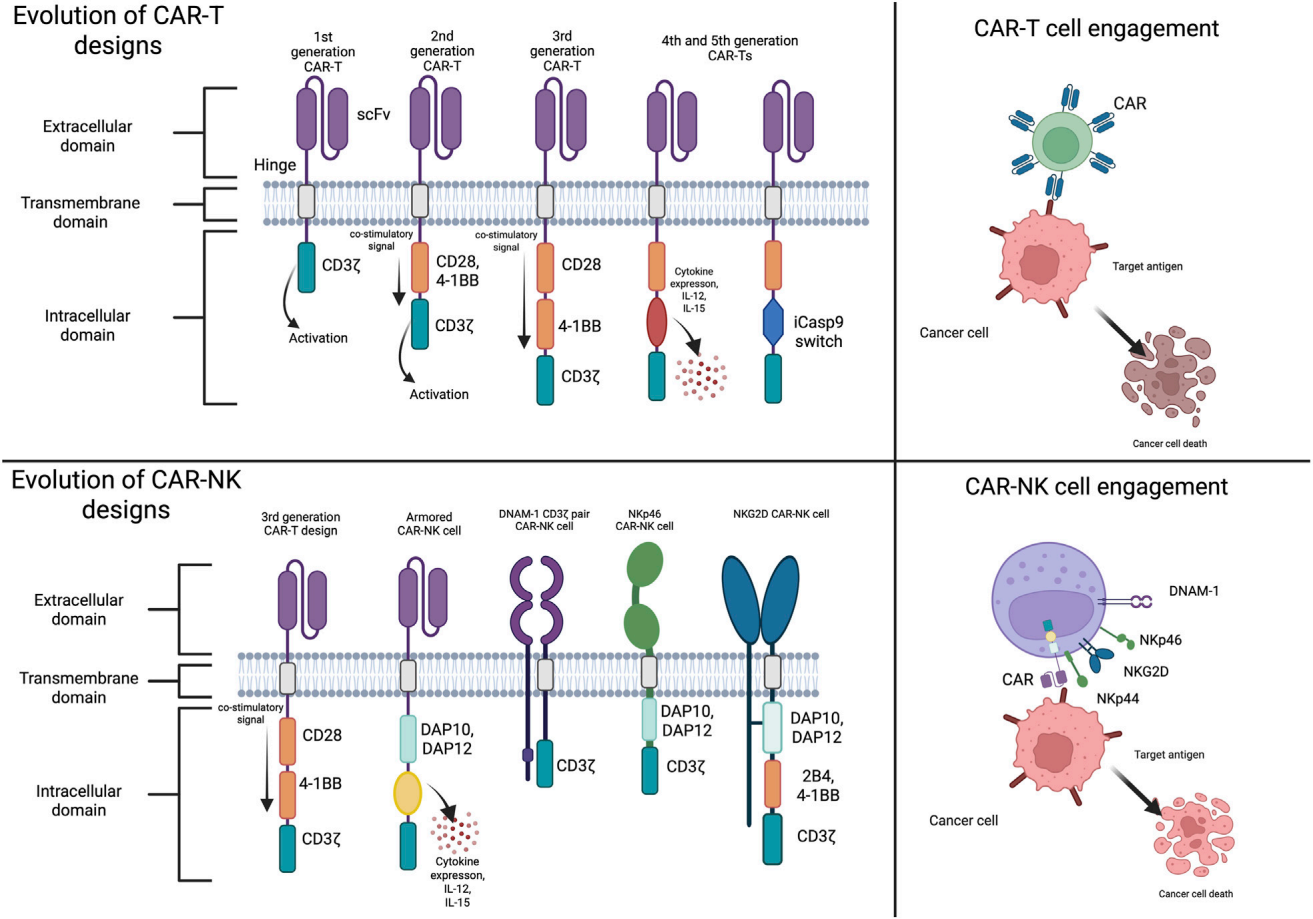
Evolution of CAR-T and CAR-NK cell designs and tumor engagement mechanisms. (Left panels) The structural development of CAR-T and CAR-NK constructs is shown, with CAR-T evolving from first-generation, CD3ζ-only, receptors to current and more advanced versions, incorporating costimulatory domains (CD28, 4-1BB), cytokine cassettes (IL-12, IL-15), or safety switches. CAR-NK designs use NK-specific signaling modules such as DAP10, DAP12, and 2B4, as well as NK-adapted receptors (DNAM-1, NKG2D, NKp46), paired with CD3ζ or NK adaptors, and include cytokine-armored designs. (Right panels) Functional comparison of CAR-T and CAR-NK cell engagement with tumor targets. Upon antigen recognition, CAR-T cells activate canonical T-cell signaling cascades leading to cytotoxicity, proliferation, and inflammatory cytokine release. Besides the CAR interaction, CAR-NK cells use a distinct receptor landscape (e.g., NKp44, NKp46, NKG2D) and NK-specific signaling adaptors to mediate rapid, MHC-independent tumor killing. Abbreviations: CAR, chimeric antigen receptor; CAR-NK, CAR-engineered natural killer cell; CAR-T, CAR-engineered T cell; CD3ζ, CD3 zeta chain; CD28, cluster of differentiation 28; 4-1BB, CD137 co-stimulatory domain; DAP10, DNAX-activation protein 10; DAP12, DNAX-activation protein 12; DNAM-1, DNAX-accessory molecule-1 (CD226); iCasp9, inducible caspase-9 safety switch; IL-12/IL-15, interleukin-12/interleukin-15; NKG2D, natural killer group 2, member D; NKp44/NKp46, natural cytotoxicity receptors; scFv, single-chain variable fragment; 2B4, SLAM family member 4 (CD244). Created in https://BioRender.com.

Collectively, these data support designing CARs that signal through NK-preferential pathways rather than directly importing T cell backbones, in order to boost efficiency. We illustrated how the structural principles and CAR backbones evolved when adapted to T-cell versus NK-cell biology in a schematic comparison of CAR-T and CAR-NK architectures and their respective effector mechanisms (Figure 1).

## 5 Sources of NK cells and CAR-NK cell engineering

A popular method of NK cell sourcing involves directly harvesting mature NK cells from either peripheral blood or umbilical cord blood (UCB) (Herrera et al., 2019). A second option, as described by Spanholtz et al., focuses on obtaining hematopoietic stem cells (HSCs) from cord blood, which are differentiated into NK cells *ex vivo* at a later stage (Spanholtz et al., 2011). As opposed to allogeneic adoptive T cell therapies, the clinical scale of expansion in allogeneic NKs is essential for providing a sufficient number of cells to exert the anti-tumor effects without the risk of increasing rates of GVHD (Baggio et al., 2017).

### 5.1 Peripheral blood-derived NK cells

Out of all available sources, peripheral blood (PB) derived NK cells are the easiest to obtain, although their use is restricted by their low transduction potential and inefficient expansion (Szmania et al., 2015).

### 5.2 Umbilical cord blood-derived NK cells

UCB-derived NK cells have been proven to generate greater proliferative capabilities (Della Chiesa et al., 2012). However, given their immature nature, their cytotoxic potential is significantly diminished compared to other sources. Compared to PB-NKs, UCB-NK cells express CD56^bright^, high density of NCR receptors and NKG2D, which are mostly involved in cytokine secretion. By contrast, adhesion molecules and receptors associated with cytotoxic killing, such as CD16, KIRs, DNAM-1, NKG2C, IL-2R and CD57 are expressed at lower levels (Tanaka et al., 2003; Luevano et al., 2012).

### 5.3 Hematopoietic stem cell-derived NK cells

HSCs allow large numbers of NKs to be collected and are more permissive to engineering enhancements (Zeng et al., 2017). CD34^+^ HSCs from the bone marrow (BM) and UCB can be used to generate NK cells, showing remarkable functionality, similar to PB-NKs in regard to cytokine generation, cytotoxic capabilities and activating potential (Luevano et al., 2014).

### 5.4 Induced pluripotent stem cell (iPSC)-derived NK cells

There are multiple possibilities of harnessing NK cells for “off the shelf” use in CAR manufacturing, such as utilizing various NK cell lines or by apheresis of iPSC-NKs (induced pluripotent stem cell-derived NK cells). In a comprehensive review, Wei et al. proposes that iPSC-derived NK cells can be generated with high clonal uniformity, a standardized manufacturing approach and enhanced genetic engineering to bolster cytotoxicity and ADCC potential. Progress in this direction was made possible by introducing 3D embryoid-body/spin-EB methods for generating CD34^+^ hematopoietic progenitors and then mature NK cells. However, challenges for successful iPSC-NK translation remain, as improving differentiation efficiency and reproducibility, gene-editing safety (off-target risks), and enhancing *in-vivo* trafficking are still elements that solicit finer tuning (Wei et al., 2025).

### 5.5 NK-92 and cell line-derived NK cell lines

There is still the possibility of utilizing cell lines, such as YT or NK-92, to engineer CAR-NKs with the desired characteristics. These cell lines represent a potentially readily available and abundant source of NK cells for immunotherapy, especially due to their ability to retain cytotoxic potential during the transduction process (Maki et al., 2001; Cheng et al., 2012). However, there are still important safety challenges when generating CAR-NKs from tumor cell lines and, often, irradiation is mandatory, which significantly affects their persistence in the host (Klingemann et al., 1996; Tang et al., 2018). Feeder cell lines can be used to expand NK cells *ex vivo*. For instance, K562 is a cell line that is MHC negative and is engineered with the purpose of generating IL-15 and IL-21 cytokines to expand and mature NK cultures (Tonn et al., 2013).

Importantly, while each NK cell source differs in cytotoxic potency, expansion kinetics, and suitability for “off-the-shelf” production, the efficiency and stability of CAR expression are determined by the gene delivery and manufacturing platform rather than the cellular origin itself (Morgan et al., 2021).

## 6 CAR-NK genetic engineering and delivery platforms

The insertion of foreign genetic material and the subsequent proliferation of NK cells present significant challenges; therefore, an appropriate and effective transfection approach is a crucial step for clinical trials (Tomanin and Scarpa, 2004). Contemporary engineering strategies, including NK-preferential co-stimulation, armoring, adaptor/switchable CARs, and multi-antigen constructs, are reviewed in recent studies and are increasingly reflected in current pipelines.

### 6.1 Manufacturing and scalability considerations

The translation of CAR-NK therapies from experimental stages to widespread clinical use requires rigorous adherence to Good Manufacturing Practice (GMP) standards governing cell handling and genetic modification to expansion, formulation, and cryopreservation. Unlike autologous CAR-T modalities, which require patient-specific manufacturing, CAR-NK therapies benefit from the feasibility of batch-based production from universal donor sources or iPSC-derived NK populations. This model enables a single manufacturing run to generate large numbers of clinical doses, with lower patient variability, shorter production time, and improves cost efficiency (Rezvani et al., 2017).

Viral vectors (lentiviruses and retroviruses) remain widely used for CAR gene integration due to their stable expression, but they come with manufacturing complexity and cost due to extensive biosafety measures needed to mitigate the risk of insertional mutagenesis (Milone and O’Doherty, 2018). Therefore, interest in alternative engineering methods has grown, particularly as NK cells present unique challenges in viral transduction.

GMP expansion of NK cells depends on regulated cytokine support, such as IL-2 or IL-15, delivered alongside feeder-cell systems. Automated and closed-system bioreactors have markedly improved scalability and manufacturing reproducibility, enabling the generation sufficient NK cells per batch for allogeneic CAR-NK platforms (Jahan et al., 2024).

### 6.2 Armored CAR-NK constructs

Fourth-generation armored CAR-NK cells are engineered to co-express self-sustaining survival signals, such as IL-15, so that they maintain proliferation, function and persistence without requiring exogenous cytokine administration.

Distinctions between IL-15–armored CAR-NK and IL-15–engineered CAR-T cells are important to note. In CAR-NK products, IL-15 primarily enhances NK-cell survival, metabolic fitness, and serial cytotoxicity through IL-15Rβ/γ signaling without provoking excessive cytokine-release toxicity, reflecting the innate regulatory checkpoints of NK cells (Christodoulou et al., 2021). By contrast, IL-15-expressing CAR-T products activate potent autocrine IL-2/IL-15 receptor pathways that can drive uncontrolled proliferation, high systemic cytokine levels, and increased risks of CRS, often requiring additional safety circuits or switch-based architecture (Hurton et al., 2016; Li et al., 2025).

Early proof-of-concept studies demonstrated that expression of membrane-bound IL-15 supports autonomous NK expansion and enhances cytotoxicity, providing a strong argument for incorporating IL-15 into efficient CAR-NK designs (Imamura et al., 2014; Liu et al., 2018). From a pharmacological perspective, these constructs reduce the need for systemic cytokine dosing, which, in turn, limits off-target immune activation and repeated infusions. IL-15 withdrawal leads to rapid NK-cell apoptosis, therefore, IL-15–armored CAR-NK cells typically achieve prolonged persistence with a low incidence of secondary toxicity (Vivier et al., 2011).

The key ability to destroy tumor cells without MHC restriction makes NK cells extremely versatile immune effectors and perfectly suited for adoptive cell therapies (Ruggeri et al., 2002; Miller et al., 2005). Furthermore, CAR-NKs lyse tumor cells, promoting apoptosis, through FasL, TRAIL, perforins/granzymes pathways and cooperation with T cells, macrophages and dendritic cells (Screpanti et al., 2001; Vivier et al., 2011). These multiple killing mechanisms complimenting the CAR interaction support broad antitumor activity and lower the risk antigen escape mechanisms (Farag and Caligiuri, 2006; Sotillo et al., 2015) and contribute to the generally favorable safety profile of NK cell therapies (Simonetta et al., 2017).

### 6.3 Advances in CAR architecture and molecular design

CAR engineering is continuously advancing to enhance receptor affinity while improving tumor selectivity, aiming to increase efficacy, as well as reduce off-target effects and antigen escape (Rodriguez-Garcia et al., 2020). One of the components that can be selectively improved is the scFv extracellular fragment. Researchers are developing dual or multi-targeting scFVs, which aim to enhance CAR antigen binding by either engaging two different epitopes on the same target, or by recognizing multiple antigens on the tumor cell (Tahmasebi et al., 2021).

Moreover, unconventional scFv fragments are currently being developed, such as nanobody derived single domain variable heavy chain (Safarzadeh Kozani et al., 2022) or fully human heavy-chain-only variable domain (FHVH), which would allow superior expression, stability and safety of smaller CAR constructs with considerably less immunogenicity than standard constructs (Lam et al., 2020). Another notion that is being developed is that of switchable CARs, where instead of directly binding tumor antigens, they target intermediary molecules—such as antibody fragments or adaptor proteins like zipFv—that, in turn, recognize the tumor. This personalized “switch” allows for precise, tunable control of the CARs specificity and function (Raj et al., 2019; Qi et al., 2020; Cao et al., 2021).

### 6.4 Viral vector-based CAR delivery

Integrating viral vectors remain a mainstay for CAR insertion in NK cells. Third-generation lentiviral and retroviral vectors support stable genomic integration and long-term CAR expression in both primary NK cells and NK-92 cell lines, building on decades of experience in gene therapy and CAR-T manufacturing (Tomanin and Scarpa, 2004; Milone and O’Doherty, 2018).

Although these platforms are thoroughly characterized from a manufacturing perspective and comply with GMP workflows in CAR-Ts, they pose unique challenges for NK cells. Primary NK cells are notably refractory to viral transduction. Compared with T cells, they express higher levels of PRRs (pattern-recognition receptors) and antiviral sensors, including TLR3, RIG-I, MDA5 and downstream MAVS-dependent pathways, which detect viral RNA and vector components (Littwitz et al., 2013; Schmidt et al., 2021; Robbins et al., 2021; Afolabi et al., 2019). Activation of these pathways reduces transduction efficiency and can trigger apoptosis or functional impairment. To further elevate transduction efficiency, PDK1 inhibitors against RIG-I-like and Toll-like receptors were used in some instances (Clark et al., 2009; Sutlu et al., 2012), even though multiple transduction processes were required. Optimization of activation status and culture conditions, vector pseudotyping, transient modulation of innate signaling are strategies that have improved NK transduction in specific settings, but more universally efficient protocols still remain in development (Sutlu et al., 2012; Schmidt et al., 2022). Cost and regulatory complexity are additional constraints. Lentiviral vector manufacture is a major cost driver in CAR-T programs, and similar considerations apply to CAR-NK models. Large-scale vector production requires specialized facilities, extensive testing and long lead times, all of which can overturn the need for cost-effective deployment of allogeneic products (Milone & O’Doherty, 2018).

Although viral vectors remain indispensable for many current products, their limitations have sparked rising interest in non-viral engineering platforms for future CAR-NK products.

### 6.5 Non-viral CAR delivery systems

Non-viral engineering platforms are promising for CAR-NK manufacturing, especially for scalable, cost-effective, and safe allogeneic applications. Transposon systems such as Sleeping Beauty and piggyBac enable stable genomic integration of large genetic material using plasmid DNA, avoiding variability and insertional mutagenesis risks associated with high-titer viral vectors (Rostovskaya et al., 2012; Matosevic, 2018; Robbins et al., 2021). piggyBac-engineered CAR-NK cells have demonstrated efficient gene transfer, robust cytotoxicity, and favorable manufacturing characteristics in preclinical models, including CAR NK-92 cells expressing NKG2D and primary CAR-NK products targeting CD73^+^ solid tumors (Wang et al., 2018; Li et al., 2020; Du et al., 2021).

Electroporation-based approaches provide a flexible, virus-free route to deliver DNA, mRNA, or ribonucleoprotein complexes. Ingegnere et al. developed a procedure that can efficiently introduce CAR and CCR7 genes via an electroporation-based plasmid DNA transfection in both human NK cell and in NK-92 lines, demonstrating that optimized plasmid DNA electroporation protocols now achieve superior transfection efficiencies in IL-2–expanded NK cells. However, short-term viability is still a concern, reportedly reaching only 50%–60% after transfection in carefully tuned systems (Ingegnere et al., 2019; Heintz and Gong, 2020; Schmidt et al., 2021; Wang et al., 2022). Multi-gene constructs delivered through DNA insertion methods allow enhanced expansion and persistence compared to mRNA-based strategies. However, harsher nuclear delivery conditions and DNA-sensing pathway activation can still affect product consistency and potency.

mRNA electroporation achieves transient CAR expression without genomic integration, making it particularly useful for early-phase, dose-escalation studies and for indications where reversible activity is desirable. Several groups have demonstrated efficient CAR-mRNA delivery into both primary NK cells and NK-92, with expression lasting a few days and maintaining potent short-term cytotoxicity (Schmidt et al., 2022; Laskowski et al., 2022). Transient mRNA CAR-NK products are more suitable to function as a built-in safety layer, enabling careful titration of exposure while platforms are optimized.

Because “off-the-shelf” CAR-NK products are allogeneic, they are inherently susceptible to host-versus-graft immune rejection driven by residual recipient T cells, NK cells, and macrophages, which can limit their persistence. In addition to gene delivery and construct stability, non-viral platforms are progressively integrating immune-editing approaches to improve the persistence of allogeneic CAR-NK cells. Recent studies show that selectively knocking down classical HLA-A/B/C and adding PD-L1 and/or HLA-E expression can prevent rejection by host T and NK cells while maintaining self-tolerance, allowing for prolonged engraftment and antitumor effects (Liu et al., 2025). A 2025 review paper highlights complementary non-viral approaches such as β2-microglobulin knockout, HLA-E overexpression, and CD47 upregulation to inhibit phagocytosis, all of which can be implemented through non-viral editing strategies (Kim, 2025).

Viral and non-viral vectors vary in efficiency, durability, and safety. Lentiviral and retroviral vectors achieve high CAR insertion rates in NK cells (∼50%–90%) and stable long-term expression, but they pose insertional mutagenesis risks and increase manufacturing complexity and costs (Schmidt et al., 2021; Dan and Kang-Zheng, 2025). Non-viral platforms like transposons (piggyBac, Sleeping Beauty) and mRNA electroporation yield variable engineering efficiencies (about 30%–70%, depending on NK source, activation, and electroporation conditions), but provide lower genotoxicity, faster production, and better scalability for allogeneic products (Ingegnere et al., 2019; Du et al., 2021; Bexte et al., 2024; McErlean and McCarthy, 2024). In comparison to viral integration, mRNA-based approaches result in transient CAR expression lasting several days, which improves safety profiles but restricts cell persistence. By contrast, transposon systems enable stable gene integration and offer lower vector-related complexity and cost (Boissel et al., 2009; Schmidt et al., 2022; Maia et al., 2024). Together, these distinctions inform platform selection for next-generation CAR-NK manufacturing and highlight complementary trade-offs in efficiency, safety, and clinical applicability.

### 6.6 Closed-system bioreactors and workflow standardization

To fully realize the promise of allogeneic CAR-NK products, advances in genetic engineering must be matched by high-end manufacturing technologies. Closed, automated bioreactor systems now enable optimal expansions of PB, UCB, or iPSC-derived NK cells in clinical settings, while maintaining phenotypic stability and cytotoxic function (Zhang et al., 2022; Moscarelli et al., 2022). The automation process reduces operator variability, lowers contamination risk and integrates well with gene-modification workflows, making them well suited for large quantity allogeneic products. Current protocols can generate around 10^9^–10^11^ NK cells per batch, sufficient to supply multiple patients from a single run, particularly when combined with optimized cryopreservation strategies (Ma et al., 2025).

## 7 Outlook on future engineering trends and solutions

The rapid advancement of CAR-NK engineering presents unique opportunities to overcome specific immune evasion mechanisms outlined earlier in this review. Below we identify actionable, evidence-supported directions that are positioned to shape the next phase of clinical translation.

### 7.1 Synthetic biology platforms for precision control

Switchable, universal and adapter-based CAR architectures allow real-time modulation of NK cytotoxicity and targeting. Peer-reviewed studies in CAR-T systems (e.g., SUPRA CARs; inducible ON-switch CARs) demonstrate feasibility, and preclinical CAR-NK adaptations are emerging. First, switchable CAR systems provide external control over NK-cell activity: “ON-switch” CARs activate only when a harmless small molecule is present, while “OFF-switch” circuits (e.g., iCasp9) allow rapid shutdown of the therapy in case of toxicity. This reversible control enhances safety and reduces off-tumor effects (Zhao et al., 2023). Second, universal CAR platforms expand targeting flexibility by decoupling antigen recognition from NK-cell activation. These systems use adapter molecules or modular components (anti-FITC CARs, SUPRA CARs) that separate recognition and signaling domains. By exchanging adapters or modules, a single CAR-NK product can be redirected to multiple tumor antigens or tuned in activity (Zhao et al., 2023; Amoozgar et al., 2025).

Future development should prioritize Boolean-logic gates and adapter systems that specifically counter antigen heterogeneity and antigen-loss mechanisms as highlighted in Sections 3.1–3.3.

### 7.2 Logic-gated CAR-NK cells for heterogeneous tumors

Dual-input AND-gate circuits, OR-gated multispecific receptors, and inhibitory NOT-gate systems show early clinical and preclinical success in enhancing selectivity and reducing off-tumor toxicity. Boolean logic–gated designs that integrate multiple antigens improve tumor specificity and reduce off-target toxicity. These CAR-NK cells can be programmed with AND, OR, and NOT gates, enabling activation only under precise antigen combinations (Zhao et al., 2023). SENTI-202 is an innovative CAR-NK therapy targeting AML. It uses two activating CARs (CD33 and FLT3) as an OR-gate to destroy leukemia cells with either antigen, countering tumor diversity. An inhibitory CAR detects EMCN, a marker on healthy stem cells, acting as a NOT-gate to prevent NK activation and protect normal bone marrow (Dos Reis et al., 2025).

Beyond SENTI-202, other types of synNotch receptors enable sequential AND-gates. These multi-input circuits requiring antigens A AND B, or A AND NOT B allow far more refined discrimination between malignant and healthy tissues than single-antigen CARs (Zhao et al., 2023; Dos Reis et al., 2025). Boolean logic gating represents a major step toward next-generation, highly selective CAR-NK immunotherapies that are particularly well-suited to counter lineage switching (Section 3.4) and mixed-antigen expression in AML and solid tumors.

### 7.3 iPSC-derived NK platforms

iPSC-derived NK cells are emerging as a transformative platform for CAR-NK therapy, offering advantages that overcome the limitations of donor-derived NK sources. By expanding indefinitely and differentiating into NK cells at scale, iPSC-NKs provide a renewable, uniform, and batch-manufacturable source of effector cells (Amoozgar et al., 2025). Early clinical studies support their feasibility, as shown by FT596, an iPSC-derived CAR-NK engineered with IL-15 support, which demonstrated antitumor activity and a favorable safety profile in CD19^+^ lymphoma (Kim, 2025). Large-scale, cryopreservable manufacturing, clonal uniformity that reduces patient variability and the ability to generate “armored” or immunologically “stealthy” CAR-NK cells makes iPSC-NKs ideal for multiplex editing to counter host rejection (Section 3.7) and to install CARs resistant to immune evasion (Amoozgar et al., 2025; Kim, 2025).

iPSC-derived CAR-NKs represent a successful design that combines manufacturing scalability with ingenious engineering, accelerating development of next-generation CAR-NK therapies.

### 7.4 Multiplex gene editing to enhance persistence and immune compatibility

Multiplex genome editing is becoming central to building CAR-NK cells that can persist in allogeneic hosts and resist immune rejection. One strategy is knocking out β2M (β-2 microglobulin), which removes polymorphic HLA-A/B/C to prevent host T-cell recognition. To avoid triggering host NK “missing-self” responses, HLA-E or HLA-G are re-expressed, which subsequently engages NK inhibitory receptors and protects the infused cells (Kim, 2025). In addition to this mechanism, CD47 overexpression blocks macrophage phagocytosis, checkpoint deletions (PD-1, TIGIT) prevent tumor-mediated suppression, and CISH knockout boosts cytokine signaling and persistence in the tumor microenvironment (Amoozgar et al., 2025; Kim, 2025). These multiplexed edits can be combined to produce immune-evasive, long-lived CAR-NK cells.

Liu et al. (2025) developed NK cells with simultaneous HLA modulation, CAR expression, and checkpoint targeting, resulting in resistance to T- and NK-cell clearance, longer persistence, and high cytotoxicity with lower inflammatory cytokines. Early clinical trials of multiplex-edited CAR-NKs show promising safety without CRS or ICANS, but extensive edits may cause toxicity, signaling the need for careful engineering.

Overall, multiplex editing can enable universal, durable, and immune-evasive CAR-NK therapies designed for broad off-the-shelf application and could be a future staple in CAR design.

### 7.5 Affinity engineering drives

Trogocytosis remains a major driver of antigen-density loss in CAR therapies, leading to reduced target availability, impaired effector function, and CAR cell fratricide. Studies in CAR-T cells have demonstrated that high-affinity scFvs promote excessive antigen extraction, leading to excessive loss of surface targets and enabling rapid immune escape (Olson et al., 2022). Conversely, lower-affinity CAR constructs preserve antigen density, reduce fratricidal interactions, and maintain durable cytotoxicity while achieving efficient synapse formation (Olson et al., 2022). Although these data stem from CAR-T systems, emerging NK-specific CAR platforms are beginning to adopt similar affinity-tuning strategies. Lower-affinity CARs may therefore allow NK cells to retain natural cytotoxic pathways, reduce tonic signaling, and limit trogocytosis-mediated resistance described in Section 3.7. Future work should optimize affinity ranges specifically for NK cells, considering NK-synapse kinetics, serial-killing behavior, and the balance between CAR-mediated and innate-mediated activation.

### 7.6 Armored CAR-NK cells against TME

The TME induces metabolic and cytokine-mediated suppression that dampens NK cell activity, driving the rationale for developing “armored” CAR-NK designs. IL-15 armoring boosts NK survival, proliferation, mitochondrial fitness, and serial killing without the toxicities seen in IL-15–enhanced CAR-T cells (Christodoulou et al., 2021). Chemokine receptor engineering (CXCR1, CXCR4, CCR7) improves trafficking and tumor infiltration both in hematological and solid tumors (Ng et al., 2019; Andreou et al., 2025). Inducing resistance to TGF-β, a major NK-suppressive cytokine, can be achieved through receptors or gene knockouts, preserving NKG2D expression, cytotoxicity, and proliferation (Christodoulou et al., 2021; Valeri et al., 2022). Other mechanisms involve suppressing the metabolic pathways, such as removing A2A receptor signaling or enhancing glycolysis to counter hypoxia and adenosine build-up (Valeri et al., 2022).

In practice, the most effective CAR-NK cells will likely combine IL-15 support, optimized chemokine homing and resistance to TGF-β or metabolic inhibition, ideally integrated at the iPSC stage for stable, uniform expression and scalable production (Andreou et al., 2025; Valeri et al., 2022).

## 8 Comparative efficacy: CAR-T vs. CAR-NK

CAR-T cell therapies are already widely available and integrated in clinical practice, especially in hematological malignancies. Since 2023, seven products have received enthusiastic approval for their efficacy in otherwise refractory disease settings (Mitra et al., 2023).

CD19^−^ and BCMA-targeted CAR-T therapies have achieved remarkable success in the treatment of B-cell neoplasms, as shown in Table 1 (June et al., 2018; Roex et al., 2020). High remission rates obtained in relapsed or refractory B cell malignancies by products like tisagenlecleucel and axicabtagene ciloleucel encouraged further development of newer and more efficient CAR constructs. Despite the ongoing popularity of engineered T cells, there are still several major drawbacks associated with these treatments. Side effects such as CRS and ICANS are still an important concern, while access is considerably limited by the high costs and complexity of the manufacturing process of personalized cells (Borgert, 2021). A sound approach to reduce manufacturing costs would involve the possibility to develop “off the shelf”, readily available engineered cells that could be used in an allogenic manner (Cutmore and Marshall, 2021). However, currently, all commercially available CAR-Ts are restricted to autologous use due to risk of GVHD.

**TABLE 1:** Commercially available CAR-T cell therapies.

CAR-T therapy (brand; generic)	Patient population (pivotal trial)	Median PFS	Median OS	CRS incidence (any; ≥Gr3)	ICANS incidence (any; ≥Gr3)	GVHD
1.Kymriah (tisagenlecleucel)	R/R B-cell ALL (pediatric/YA; ELIANA trial)	18.4 months	2 years OS 43.6%	77% (≥Gr3: 48%)	71% (≥Gr3: 22%)	Not available (autologous)
2.Yescarta (axicabtagene ciloleucel)	R/R large B-cell lymphoma (adult; ZUMA-1)	48 months 41.8%	5 years OS 69%	94% (≥Gr3: 13%	94% (≥Gr3: 31%)	N/A (autologous)
3.Tecartus (brexucabtagene autoleucel)	R/R mantle cell lymphoma (adult; ZUMA-2)	N/A	Cohort 1 5 years OS 39%	91% (≥Gr3: 18%)	81% (≥Gr3: 37%)	N/A (autologous)
4.Breyanzi (lisocabtagene maraleucel)	R/R large B-cell lymphoma (adult; TRANSCEND)	N/A	N/A	54% (≥Gr3: 3.2%)	31% (≥Gr3: 10%)	N/A (autologous)
5.Abecma (idecabtagene vicleucel)	R/R multiple myeloma (adult; KarMMa trial)	13.3 months	N/A	84% (≥Gr3: 4%)	18% (≥Gr3: 4%)	N/A (autologous)
6.Carvykti (ciltacabtagene autoleucel)	R/R multiple myeloma (adult; CARTITUDE-1)	34.9 months	Not reached	95% (≥Gr3: 4%)	23% (≥Gr3: 3%)	N/A (autologous)
7.Aucatzyl (obecabtagene autoleucel)	R/R B-cell ALL (adult; FELIX trial)	N/A	N/A	75% (≥Gr3: 3%)	24% (≥Gr3: 7%)	N/A (autologous)

All approved CAR-T, products are autologous (patient-derived T cells); therefore, no GVHD, has been observed in these trials. In pediatric/young adult ALL (Kymriah), CRS, and ICANS, rates were high due to heavy disease burden. Among CD19-targeted products for lymphoma, Yescarta (axicel) showed higher severe neurotoxicity than Breyanzi (liso-cel), reflecting different CAR, designs (CD28 vs. 4-1BB, costimulation). BCMA-directed CAR-T, for myeloma (Abecma, Carvykti) caused CRS, in the majority of patients (often grade 1–2), while ICANS, was less frequent (∼20% or less). Aucatzyl (obe-cel) for adult ALL, achieved notably low rates of ≥Grade 3 CRS (3%) and ICANS (7%) in its trial, partly due to its fractionated dosing strategy (Maude et al., 2018; Neelapu et al., 2017; Lee et al., 2019; Wang et al., 2020; Abramson et al., 2020; Munshi et al., 2021; Berdeja et al., 2021; Locke et al., 2024; Roddie et al., 2024).

### 8.1 Advantages and disadvantages of CAR-T/CAR-NK therapies

This review has identified and summarized the primary advantages and disadvantages of these immunotherapies, which are outlined in Table 2.

**TABLE 2:** Comparison between CAR-T and CAR-NK immunotherapies.

Feature	CAR-T cells	CAR-NK cells
Source	Autologous only (patient-derived T cells); risk of GVHD so far prevents allogeneic use	Multiple allogeneic sources possible (peripheral blood, cord blood, HSCs, iPSC-NKs, NK cell lines); minimal GVHD risk
Manufacturing	Complex, high costs, time-consuming; limited accessibility	Easier, scalable with “off-the-shelf” production; easier availability, especially for rapidly progressive disease
Clinical use	Approved in hematological malignancies (CD19 and BCMA targets) with high remission rates; 7 FDA-approved products	Still investigational; phase I/II trials show safety and early efficacy in hematological malignancies (e.g., CD19, BCMA, CD33, NKG2D)
Efficacy	Long-term persistence and high response rates in B-cell malignancies	Active in both hematological and solid tumors; encouraging CRs/PRs, but persistence and durability still limited
Safety/toxicity	High risk of CRS and ICANS; prolonged B-cell aplasia (on-target/off-tumor effect)	Lower toxicity; rare CRS/ICANS; no GVHD; limited persistence reduces long-term on-target/off-tumor effects; safety switches (e.g., iCasp9) can be integrated
Tumor escape	Susceptible to antigen-negative clones, mutations, lineage switch, trogocytosis, epitope masking	Less susceptible to MHC-I loss; still vulnerable to antigen downregulation and trogocytosis (though may sometimes enhance NK functions)
Persistence	Strong persistence *in vivo*, sometimes leading to durable remissions	Shorter lifespan; persistence can be improved with cytokine support (IL-15, IL-12, IL-18), armored CAR-NKs, or memory NK induction
Cytotoxic mechanisms	Mainly CAR-directed killing via perforin/granzymes	Multiple innate killing pathways (CAR-directed + FasL, TRAIL, ADCC, cytokine production), enhancing tumor clearance and reducing relapse risk
Accessibility	Personalized; limited patient access due to cost/logistics	Scalable, universal product potential; faster access, especially critical for aggressive disease

Advantages and disadvantages of CAR-T, versus CAR-NK, cells. This table addresses the issues of cell sources, manufacturing procedures, clinical applicability, efficiency and safety concerns, tumor escape risks, long term persistence, killing mechanisms and accessibility limitations.

### 8.2 Safety profile and toxicity

With their unique ability to locate and destroy target cells, NK cells make ideal candidates for CAR augmentation. Notably, CAR-expressing NK cells possess a safer therapeutic profile than CAR-T cells in clinical settings, and multiple clinical trials have demonstrated that NK cell immunotherapy is a viable alternative to CAR-T therapy (Becker et al., 2016). For instance, phase I/II trials have shown that allogeneic NK cell administration is well tolerated and does not induce GVHD or other severe adverse events, highlighting NK cells as general CAR drivers independent of autologous cells (Sakamoto et al., 2015; Ciurea et al., 2017). Additionally, newer constructs incorporate iCasp9 (inducible caspase-9) “safety switch” (Figure 1), which triggers programmed death of CAR-NK cells in the event that severe toxicity occurs (Nowakowska et al., 2018; Liu et al., 2020). This safety switch is a necessary control mechanism as even stronger “armored” CAR-NK cells are being developed, which could also incur greater side effects (Włodarczyk and Pyrzynska, 2022).

A major drawback of CAR-T therapy is the persistent on-target/off-tumor effects, such as CD19 CAR-T cells causing prolonged B-cell aplasia. In contrast, the limited lifespan of CAR-NK cells in circulation reduces such risks. Another possible complication is trogocytosis. This is a process in which immune cells express membrane proteins “nibbled” from target cells, which further leads to depletion of target cells antigens and, also, to cellular fratricide between CAR-T products (Miao et al., 2021). In CAR-NK cells, however, there is limited available data on this phenomenon and extensive research is already being carried out. It was observed that trogocytosis-mediated signaling can induce different types of behavior in NK cells (Vanherberghen et al., 2004; Reed et al., 2021). In some cases, NK functionality can be enhanced through trogocytosis-mediated acquisition of chemokine receptors such as CCR5, CXCR4 or CCR7 (Marcenaro et al., 2009; Somanchi et al., 2012; Vo et al., 2022) which improves homing to lymph nodes (Somanchi et al., 2012) or TYRO3, which boosts proliferation properties and effector functions (Lu et al., 2021). Potential negative effects of trogocytosis include acquisition of immunosuppressive proteins such as PD-1 (Gonzalez et al., 2021; Hasim et al., 2022), which significantly reduce their cytotoxic potential. To counter these effects, antibodies blocking CD9 were used *in vitro* with varying degrees of success in restoring antitumor efficacy (Gonzalez et al., 2021).

Additionally, depletion of target antigens due to trogocytosis has been observed in both CAR-T and CAR-NK therapies (Hamieh et al., 2019; Li et al., 2022; Schoutrop et al., 2022; Camviel et al., 2022). Antigen density is critical for CAR cell function, therefore potential downregulation or loss through trogocytosis can be detrimental to the effectiveness of the therapy and facilitates tumor evasion. Strategies to mitigate this complication involve adjusting the affinity of the CAR for its cognate antigen. Recent papers highlight that lower affinity CAR constructs might be able to reduce the incidence of trogocytosis without compromising efficiency (Olson et al., 2022) and further optimizing this approach might contribute significantly to limiting antigen loss and, therefore, their overall performance and persistence (Singh et al., 2020).

### 8.3 Persistence

With our current understanding of NK cell behavior, we know that they are less likely to show long term persistence as a shortened lifespan can affect overall efficiency in clinical trials (Bachanova et al., 2010; Shaffer et al., 2016; Nguyen et al., 2019). Much of the progress that has been made recently in improving NK therapy performance has been related to their cytotoxic potential. However, efforts are underway to increase NK persistence as well by engineering cells with immunostimulatory cytokines (Liu et al., 2018; Liu et al., 2020; Christodoulou et al., 2021; Du et al., 2021; Teng et al., 2022; Cichocki et al., 2022a; Cichocki et al., 2022b). Liu et al. introduced, in a clinical study, IL-15 engineered CD19-directed CAR-NK cells in relapsed/refractory hematological malignancies with moderate success (Liu et al., 2020). It was also demonstrated that exposure to IL-12, IL-15, IL-18 leads to promotion of memory NK cells, which are more persistent and efficient against tumor cells (Gang et al., 2020). This technique has proved itself in CAR-T therapies, where addition of immunomodulatory cytokines improved response, persistence and created armored CARs models (Yeku and Brentjens, 2016; Deng et al., 2020; Zhang et al., 2021). Recent evidence also suggests that chronic engagement of inhibitory KIRs contributes to functional exhaustion and reduced persistence of CAR-NK cells *in vivo*. This novel strategy proposes that inhibitory CAR (iCAR) targeting KIRs can convert an inhibitory signal into an activating one, thereby restoring metabolic fitness, enhancing proliferation, and improving tumor control in preclinical models (Li et al., 2022). Furthermore, addition of inducible promoters, which become active after recognizing tumor antigens or specific cellular signaling pathways can help increase functionality and safety of CAR therapies.

One of the most important factors that can alter NK cell persistence in patients undergoing treatment is the lymphodepleting regimen administered before cell infusion. To date, insights into the role of lymphodepletion in CAR-NK therapy have been based on CAR-T research, where this process proved its therapeutic efficacy (Amini et al., 2022). Typical regimens associate fludarabine and cyclophosphamide before CAR-NK infusion to prevent NK cell rejection and modulate the immunosuppressive tumor microenvironment (Xie et al., 2020). Additionally, a strategy utilizing a CD52-targeting monoclonal antibody in combination with a CD52-knockout CAR-T cell product has been explored and can be administered alongside cellular infusion (Caldwell et al., 2021), but, whether this strategy is applicable to CAR-NK cells, remains unknown so far. Currently, there are no clinical trials that directly compare lymphodepleting regimens in CAR-NK therapies, making this an area requiring further investigation. This need for research is even more pronounced when considering CAR-NK applications in solid tumors, where the value of lymphodepletion remains a topic of debate due to concerns that immune suppression may negatively impact endogenous anti-tumor immune responses.

## 9 Clinical trials and translational insights

CAR-NK clinical trials demonstrated veritable traction in the last few years for hematological malignancies. While a few CAR-NK studies were initiated in the pre-COVID era, most of the phase I/II studies that harness the power of NK cells started from 2021 onward.

Depending on the hematological cancer that was addressed, cellular targeting was approached differently. For instance, in relapsed/refractory B-cell malignancies, including NHL and ALL, CD19 was a marker of choice for most CAR-NK designs, while in multiple myeloma, BCMA (B cell maturation antigen) was the preferred target (Supplementary Table S1).

Most of the trials are active, in recruitment or terminated prematurely, but one is concluded and final/partial results were presented. One important element to note is that, currently, there are no studies that compare CAR-NK cells and CAR-T cells in a head-to-head trial, which might make the choice of therapy a more difficult one for the attending physician, in the long run.

In a phase I/II study conducted by a MD Anderson research group in 2017–2024, cord-blood derived NK cells were extracted and modified with a CD19^−^CD28-zeta-2A-iCasp9-IL15 structure to target cancer cells in relapsed/refractory CD19^+^ B cell malignancies, including lymphomas, chronic lymphocytic leukemia and, also, acute lymphoblastic leukemias (proportions not specified). All 49 enrolled subjects were split into four study groups, and after a lymphodepleting regimen based on Fludarabine, Cyclophosphamide and Mesna, CAR-NK cells were infused at different doses (Liu et al., 2020; Marin et al., 2024). Responses were assessed across the groups at the 30-day and 100-day time mark, respectively. Out of 49 subjects, 19 (38.8%) achieved complete response and 4 (8.1%) a partial response. Adverse events were monitored in the first 40 days, and amongst the most reported were infections (10/49). CNS manifestations were relatively mild and uncommon (6/49) and CRS was seldomly reported and with low grade manifestations (Liu et al., 2020; Marin et al., 2024). These findings suggest that CAR-NK cells are capable of inducing remissions in advanced hematologic cancers, all while maintaining a commendable safety profile, in advantage of their T cell counterparts.

The results of the other clinical trials are highly anticipated, as they could bring further insight into key elements that could improve persistence and effectiveness in CAR-NK cells.

### 9.1 Limitations of CAR-NK therapy

Despite ongoing progress, CAR-NK therapies face several challenges that currently limit their durability and potential. First, *in vivo* persistence of CAR-NK cells is typically shorter than that observed with CAR-T cells, with detectable CAR-NK cells often declining within weeks in the absence of cytokine support (Liu et al., 2020; Romee et al., 2016). This is indicative of the intrinsic lifespan and homeostatic demands of NK cells, which may consequently limit the persistence of antitumor responses. Second, host immune clearance, including rejection by residual T cells, NK cells, and macrophages, can eliminate infused CAR-NK cells, particularly in allogeneic settings without adequate lymphodepletion or immunomodulation (Laskowski et al., 2022; Jørgensen et al., 2025). Third, NK cells show variable trafficking and infiltration into solid tumors, where dense stroma, hypoxia, and immunosuppressive cytokines such as TGF-β limit NK cytotoxicity (Andreou et al., 2025). Finally, large-scale, standardized manufacturing pipelines remain under development, and differences among NK sources contribute to variability in phenotype and functional potency (Cichocki et al., 2022a; Cichocki et al., 2022b).

### 9.2 Emerging strategies to address CAR-NK limitations

Approaches to improve CAR-NK persistence include IL-15 armoring and induction of memory-like NK phenotypes described in preclinical models (Ma et al., 2022). To mitigate host-mediated clearance, current strategies under investigation in NK-cell therapy include the engineering of “do not eat me” signals such as CD47, overexpression of HLA-E, and gene editing to address mismatched HLA alleles (Laskowski et al., 2022). Enhancing solid tumor penetration may be achieved either by chemokine receptor engineering (CXCR4, CXCR1) to improve homing, or by altering the tumor microenvironment for improved immune infiltration (Andreou et al., 2025). Also, iPSC-derived CAR-NK platforms support reproducible, scalable, and standardized manufacturing for off-the-shelf application, helping to address variability in NK-cell source and to simplify production (Jørgensen et al., 2025).

### 9.3 Quantitative clinical benchmarks

To substantiate differences in clinical performance, we summarize commonly reported metrics from representative CAR-T and CAR-NK trials in Table 3, including overall survival, adverse event rates, expansion/persistence kinetics, and manufacturing costs. Values reflect early-phase CAR-NK studies and late-phase/approved CAR-T programs in B-cell malignancies; individual trials may deviate from these ranges.

**TABLE 3:** Comparative characteristics of CD19/BCMA CAR-T and CD19 CAR-NK therapies in B-cell malignancies.

Trial comparisons	CAR-T cells (CD19/BCMA; approved/late-phase)	CAR-NK cells (CD19; early-phase)	Key references
Clinical maturity	Multiple pivotal phase II trials with >100 pts each; several products approved in LBCL and MM; 2–5+ year follow-up showing durable remissions.	Early phase I/II trials only; typical sample size ∼11–40 pts; no regulatory approvals yet; follow-up usually ≤1–2 years.	Neelapu et al. (2017), Berdeja et al. (2021), Jørgensen et al. (2025)
Trial design and limitations	Pivotal trials are mostly single-arm but large, with clearly defined r/r LBCL or MM cohorts; increasing number of randomized/comparative CAR-T studies; long-term outcome data available.	All published CAR-NK trials are single-arm, non-randomized, and small; often include mixed B-cell malignancies (NHL/CLL/ALL) in the same protocol; no head-to-head CAR-T vs. CAR-NK trials; durability and optimal dosing remain uncertain.	Jørgensen et al. (2025), Liu et al. (2020), Marin et al. (2024)
Typical patient population	Heavily pre-treated r/r LBCL or MM (often ≥3 prior lines including rituximab/PI/IMiD/ASCT); high-risk and chemo-refractory cohorts.	Heavily pre-treated CD19^+^ B-cell malignancies (NHL, CLL, ALL); median 3–11 prior lines; often ineligible or high-risk for ASCT or autologous CAR-T.	Neelapu et al. (2017), Berdeja et al. (2021), Liu et al. (2020), Marin et al. (2024)
Overall response/CR rates	LBCL (axi-cel, ZUMA-1): ORR ∼82%–83%, CR ∼54–58%. MM (cilta-cel, CARTITUDE-1): ORR ∼98%, sCR ∼80% in heavily pre-treated MM.	CD19 CAR-NK (Liu et al., 2020, 11 pts): ORR 73%, CR 64%. IL-15-armoured UCB CAR-NK (Marin et al., 2024, 37 evaluable pts): day-30 ORR ∼49%; 1-year OS ∼68%. Overall, response rates are encouraging but generally below best-in-class CAR-T benchmarks and with less mature durability data.	Neelapu et al. (2023), Berdeja et al. (2021), Liu et al. (2020), Marin et al. (2024)
CRS	Any-grade CRS very common (≈80%–90% depending on product); grade ≥3 CRS ∼10%–20%; frequent need for tocilizumab and/or steroids.	In the leading CD19 CAR-NK trials, CRS has been rare or absent: Liu et al. (2020) and Marin et al. (2024) reported no CRS of any grade; other CAR-NK programs show only low-grade CRS.	Neelapu et al. (2017), Berdeja et al. (2021), Liu et al. (2020), Marin et al. (2024), Lei et al. (2025)
ICANS/neurotoxicity	ICANS in ∼20%–60% of patients across major products; grade ≥3 ICANS in ∼10%–30% in some cohorts; movement/neurocognitive AEs in some BCMA CAR-T programs.	No ICANS or other significant neurotoxicity reported in Liu et al., (2020) and Marin et al. (2024) CD19 CAR-NK cohorts; overall neurotoxicity appears markedly lower, though total patient numbers and follow-up remain limited.	Neelapu et al. (2017), Berdeja et al. (2021), Liu et al. (2020), Marin et al. (2024)
*In vivo* persistence	CAR-T cells can persist months to years; long-term follow-up shows ongoing CAR-T detection and remission ≥5–10 years in a subset of patients.	CAR-NK cells typically persist for weeks to a few months; IL-15-armoured constructs extend persistence, but long-term engraftment is uncommon; durability of responses is still being defined.	Melenhorst et al. (2022), Neelapu et al. (2023), Berdeja et al. (2021), Liu et al. (2020), Marin et al. (2024)
Manufacturing model	Autologous, patient-specific; apheresis → individualized manufacturing → release testing; each batch serves one patient; requires high-complexity GMP infrastructure and cold-chain logistics.	Allogeneic “off-the-shelf” from cord blood, peripheral donors, iPSC-NK or NK-92 cell lines; a single run can generate many doses; cryopreserved inventory enables decoupling of manufacturing and treatment time.	Rezvani et al. (2017), Liu et al. (2020), Marin et al. (2024), Jørgensen et al. (2025)
Scalability and time-to-infusion	Scalability constrained by patient-by-patient production and vector capacity; time from apheresis to infusion typically several weeks.	Closed, automated bioreactors plus donor/iPSC platforms can generate ∼10^9^–10^11^ NK cells per batch and support multi-dose inventories; once released, off-the-shelf products could in principle shorten time-to-treatment to days.	Marin et al. (2024), Jørgensen et al. (2025)
Cost	High costs driven by autologous workflow, intensive QC, and viral vector manufacture; reflected in current high list prices for approved CAR-T products.	Projected lower costs through batch allogeneic manufacturing and potential non-viral approaches, but real-world cost and reimbursement for CAR-NK remain to be defined because products are not yet licensed.	Jørgensen et al. (2025)
Key evidence gaps	In the particular CD19/BCMA malignancies, long-term safety and efficacy are relatively well established Remaining gaps include optimizing earlier-line use, retreatment, and solid tumor indications for CAR-T models	Major gaps include small, non-randomized cohorts; no head-to-head CAR-T vs. CAR-NK trials; limited follow-up; and uncertainty around optimal dosing, lymphodepletion, and re-infusion strategies.	Marin et al. (2024), Jørgensen et al. (2025)

This table compares CAR-T, and CAR-NK, therapies in terms of clinical performance, toxicity, persistence, manufacturing, scalability, and evidence limitations. CAR-T, data come from large phase II, trials (e.g., ZUMA-1, CARTITUDE-1) with extensive follow-up, while CAR-NK, data are mainly from smaller, early-phase studies (e.g., Liu et al., 2020; Marin et al., 2024) with less long-term data. Key differences include efficacy, safety, and pharmacological features, with major CAR-NK, challenges being short persistence, lack of randomized trials, and uncertainties about dosing, lymphodepletion, and durability. The table clarifies each platform’s strengths, challenges, and references supporting each area.

Abbreviations: ALL, acute lymphoblastic leukemia; ASCT, autologous stem-cell transplantation; BCMA, B-cell maturation antigen; CB, cord blood; CLL, chronic lymphocytic leukemia; CR, complete response; CRS, cytokine release syndrome; G3+, grade ≥ 3; ICANS, immune effector cell–associated neurotoxicity syndrome; IL, interleukin; iPSC, induced pluripotent stem cell; LBCL, large B-cell lymphoma; MM, multiple myeloma; NK, natural killer; ORR, overall response rate; OS, overall survival; r/r, relapsed/refractory; sCR, stringent complete response.

## 10 Conclusion

CAR-NK cells are emerging as a flexible and advanced type of immunotherapy. This review proposes an “evasion-to-solution” framework that links specific tumor immune evasion mechanisms with engineering strategies such as multi-antigen targeting, affinity tuning, NK-specific signaling domains, cytokine armoring, HLA editing, and iPSC platforms. These advances help CAR-NK cells counter antigen escape, antigen loss, and tumor microenvironment suppression. Although this review mainly focused on hematologic malignancies, similar engineering principles are applied to solid tumors. NKG2D CAR-NK cells have shown improved cytotoxicity and early signs of safety in metastatic colorectal cancer (Xiao et al., 2019; Li et al., 2025; Wang et al., 2025), while iPSC-derived MUC1-CAR-NK cells selectively eliminate MUC1^+^ oral tongue carcinoma with minimal toxicity (Lin et al., 2024). Their activity, however, remains limited by microenvironmental barriers including hypoxia, adenosine signaling, TGF-β, and suppressive myeloid cells (Zhang et al., 2024). Future priorities include improving persistence, enhancing trafficking into solid tumors, refining NK-specific signaling, standardizing manufacturing, and conducting comparative trials against CAR-T therapies. Addressing these gaps will accelerate the translation of CAR-NK cells into widely applicable treatments across both blood and solid cancers.

## 11 Limitations

The aim of the review is to integrate recent data regarding the complex structure of CAR-NK cells, current clinical studies on hematological malignancies and future directions of this therapy. In addition, CAR-NK therapies directed against solid tumors or non-oncological diseases were not included in our study. Due to the limited space and the abundance of literature in this rapidly evolving field, we regret that we were unable to include all the relevant studies in this review and apologize to any authors whose work may have been omitted.
