# Supplementary material for: Engineering immunity with CAR-NK cells: advancing the frontiers of cancer immunotherapy
Source: Front Pharmacol. 2025 Dec 19;16:1738558. doi: 10.3389/fphar.2025.1738558 (PMC12758028; doi:10.3389/fphar.2025.1738558)
Supplement: Supplementary file 1 [file Supplementaryfile1.docx]

**Supplementary Table 1. CAR-NK cells in clinical studies**

| **NO. NCT** | **Other**  **Name/ID**  **Numbers** | **Status** | **Start**  **Date** | **Phase** | **Disease** | **Target** | **Sponsor locations** | **NK**  **source** |
| --- | --- | --- | --- | --- | --- | --- | --- | --- |
| NCT00995137 | NKCD19  R01CA113482  NCI-2011-  01226 | Completed in May 2013 | October  2009 | I | B-Lineage Acute  Lymphoblastic  Leukemia | CD19 | St. Jude Children’s Research  Hospita | PB-NK |
| NCT01974479 | NKCARCD19 | Suspended | September  2013 | I | B-Lineage Acute  Lymphoblastic  Leukemia | CD20 | National University Health  System, Singapore | PB-NK |
| NCT03056339 | 2016-0641  NCI-2018-  01221 | Completed | June 21,  2017 | I and  II | B Lymphoid  Malignancies | CD19 | M.D. Anderson Cancer  Center | UCB-NK |
| NCT03824964 | CD19/CD22  CAR NK-  BJZL-01 | Unknown | February 1,  2019 | I | Relapsed or Refractory  B Cell Lymphoma | CD19/  CD22 | Allife Medical Science and  Technology Co., Ltd | Unknown |
| NCT03692767 | CD22 CAR  NK-BJZL-01 | Unknown | March  2019 | I | Relapsed and Refractory  B Cell Lymphoma | CD22 | Allife Medical Science and  Technology Co., Ltd. | Unknown |
| NCT03690310 | CD19 CAR  NK-BJZL-01 | Unknown | March  2019 | I | Relapsed and Refractory  B Cell Lymphoma | CD19 | Allife Medical Science and  Technology Co., Ltd. | Unknown |
| NCT04245722 | FT596-101 | Terminated | March 19,  2020 | I | B-Cell Lymphoma,  Chronic Lymphocytic  Leukemia | CD19 | Fate Therapeutics | iPSC-NK |
| NCT04623944 | NKX101-101 | Active, not recruiting | September  21, 2020 | I | Adults With AML or  MDS | NKG2D | Nkarta Inc. | PB-NK |
| NCT05215015 | IBR733-T01  WX-IBR-7 | Unknown | November  30, 2020 | I | Acute Myeloid  Leukemia | CD33/  CLL1 | Wuxi People’s Hospital | Unknown |
| NCT04639739 | CAR NK for  NHL | Unknown | December  17, 2020 | I | Relapsed or Refractory  B Cell Non-Hodgkin  Lymphoma | CD19 | Xinqiao Hospital of  Chongqing | Unknown |
| NCT04747093 | ITNK-2021 | Unknown | January 29,  2021 | I and II | B Cell Malignancies | CD19 | Nanfang Hospital of  Southern Medical  University | Induced-T  Cell  Like NK  Cells |
| NCT04796675 | CAR-NK-  CD19 cells | Unknown | April 10,  2021 | I | B Lymphoid  Malignancies | CD19 | Wuhan Union Hospital,  China | CB |
| NCT04887012 | IR2021002168 | Unknown | May 1,  2021 | I | Refractory or Relapsed  B-cell Non Hodgkin  Lymphoma | CD19 | Second Afﬁliated Hospital,  School of Medicine,  Zhejiang University | PB-NK |
| NCT05020678 | NKX019-101 | Active, not recruiting | August 20,  2021 | I | Adults With B-cell  Cancers | CD19 | Nkarta Inc. | PB-NK |
| NCT05008536 | BCMA NK for  MM | Unknown | October 1,  2021 | I | Relapsed or Refractory  Multiple Myeloma | BCMA | Xinqiao Hospital of  Chongqing | UCB-NK  and CB-NK |
| NCT05247957 | CARNK-001 | Terminated | October  13, 2021 | I | Relapsed or Refractory  Acute Myeloid  Leukemia | NKG2D | Hangzhou Cheetah Cell  Therapeutics Co., Ltd | UCB-NK |
| NCT05008575 | CD33 CAR  NK-AML | Unknown | December  23, 2021 | I | Relapsed or Refractory  Acute Myeloid  Leukemia | CD33 | Xinqiao Hospital of  Chongqing | Unknown |
| NCT05379647 | NK-002 (QN-  019a) | Unknown | November  4, 2021 | I | B-Cell Malignancies | CD19 | Zhejiang University | iPSC-NK |
| NCT05182073 | FT576-101 | Active, not recruiting | November  24, 2021 | I | Multiple Myeloma | BCMA | Fate Therapeutics | iPSC-NK |
| NCT05110742 | 2021-0526 | Recruiting | June 30,  2022 | I and  II | Relapse or Refractory  Hematological | CD5 | M.D. Anderson Cancer  Center | CB-NK |
| NCT05092451 | 2021-0386 | Recruiting | August 1,  2022 | I and  II | Relapse or Refractory  Hematological  Malignances | CD70 | M.D. Anderson Cancer  Center | CB-NK |
| NCT05336409 | CNTY-101-  111-01 | Active, not recruiting | December  2022 | I | Relapsed or Refractory  CD19-Positive B-Cell  Malignancies | CD19 | Century Therapeutics, Inc. | iPSC-NK |
| NCT05563545 | XB-NK-1003 | Completed | July 2022 | I | Relapsed/​Refractory Acute Lymphoblastic Leukemia | CD19 | Shanghai Simnova Biotechnology Co.,Ltd. | Unknown |
| NCT05472558 | 2022-0496 | Recruiting | July 2022 | I | B-cell Non Hodgkin Lymphoma | CD19 | Second Affiliated Hospital, School of Medicine, Zhejiang University | CB-NK |
| NCT06827782 | ID2024482 | Enrolling by invitation | March 2025 | I | Refractory/recurrent Central Nervous System Lymphoma | CD19 | Second Affiliated Hospital, School of Medicine, Zhejiang University | CB-NK |
| NCT06909474 | PBC083 | Recruiting | March 2025 | I | Relapsed/refractory T T-ALL/Lymphoma | CD5 | Chongqing Precision Biotech Co., Ltd | Unknown |
| NCT06696846 | 2024-0992 | Not yet recruiting | December 2024 | I | Relapsed/refractory T-cell Lymphoma  Relapsed/refractory Acute Myeloid Leukemia | CD70 | Second Affiliated Hospital, School of Medicine, Zhejiang University | CB-NK |
| NCT05645601 | CAR-NK-JD010 | Unknown | December 2024 | I | r/r B-cell Malignancies | CD19 | Affiliated Hospital to Academy of Military Medical Sciences | Allogenic NK |
| NCT06307054 | SHSY-CAR-NK-AML01 | Recruiting | March 2024 | I | Relapsed/Refractory AML | CLL-1 | Shanghai General Hospital, Shanghai Jiao Tong University School of Medicine | Autologous/allogenic NK cells |
| NCT05410041 | BRYY-IIT-LCYJ-2022-003 | Unknown | May 2022 | I | Relapsed/Refractory B cell malignancies | CD19 | Beijing Boren Hospital | Unknown |
| NCT06690827 | PBC065 | Recruiting | November 2024 | I | AML (Acute Myeloid Leukemia)  BPDCN (blastic Plasmacytoid Dendritic Cell Neoplasm) | CD123 | Chongqing Precision Biotech Co., Ltd | Unknown |
| NCT06707259 | 2024（1369） | Recruiting | December 2024 | I | Refractory/Relapsed B-cell NHL | CD19 | Second Affiliated Hospital, School of Medicine, Zhejiang University | CB-NK armored IL-10 |
| NCT05987696 | QH-TJ-01 | Not yet recruiting | August 2023 | I | AML, Adult  Minimal Residual Disease | CD33/CLL1 dual CAR-NK cells | Institute of Hematology & Blood Diseases Hospital, China | Unknown |
| NCT06027853 | QH-CLL1-01 | Recruiting | September 2023 | I | AML | CLL1 | Zhejiang University | Unknown |
| NCT06006403 | PBC050 | Recruiting | August 2023 | I/II | R/R AML  BPDCN | CD123 | Chongqing Precision Biotech Co., Ltd | Unknown |
| NCT03940833 | AsclepiusTCG02 | Unknown | May 2019 | I/II | R/R Multiple Myeloma | BCMA | Asclepius Technology Company Group (Suzhou) Co., Ltd. | NK92 |
| NCT06242249 | 74932 | Not yet recruiting | April 2024 | I/II | R/R MM | BCMA | Shahid Beheshti University of Medical Sciences | Unknown |
| NCT06631040 | 75358 | Not yet recruiting | December 2024 | I/II | B-ALL | CD19 | Shahid Beheshti University of Medical Sciences | CB-NK |
| NCT06464861 | 2024 (0673) | Recruiting | June 2024 | I | Primary Mediastinal B cell lymphoma (PMBCL)  DLBCL  MCL | CD19 | Second Affiliated Hospital, School of Medicine, Zhejiang University | Unknown |
| NCT04623944 | NKX101-101 | Active, not recruiting | September 2021 | I | AML  MDS  R/R AML  R/R MDS | NKG2D ligands | Nkarta, Inc. | Allogenic |
| NCT05667155 | 2022-088 | Recruiting | December 2022 | I | B cell NHL | CD19/CD70 | Second Affiliated Hospital, School of Medicine, Zhejiang University | CB-dualCARNK19/70 |
| NCT06334991 | QUILT-106 | Recruiting | August 2024 | I | CD19+ and CD20+ R/R B-cell NHL | CD19 | ImmunityBio, Inc. | NK-92 cell line |
| NCT05618925 | QUILT-3.092 | Recruiting | November 2024 | I | R/R B-cell NHL | CD19 | ImmunityBio, Inc. | NK-92 cell line |

**Supplementary Table 1.** This table summarizes registered clinical trials of CAR-NK cell therapies sourced from ClinicalTrials.gov. Each entry includes the trial identification number (NCT ID), study phase, disease indication, target antigen(s), trial sponsor, geographic location, cell source where it is provided (e.g., peripheral blood NK cells, cord blood NK cells, iPSC-derived NK cells, NK-92 cell line), and current recruitment status. The table highlights the diversity and rapid expansion of CAR-NK cell platforms, and targeted malignancies across early-phase studies.
